# Powerful fermentative hydrogen evolution of photosynthate in the cyanobacterium *Lyngbya aestuarii* BL J mediated by a bidirectional hydrogenase

**DOI:** 10.3389/fmicb.2014.00680

**Published:** 2014-12-10

**Authors:** Ankita Kothari, Prathap Parameswaran, Ferran Garcia-Pichel

**Affiliations:** ^1^School of Life Sciences, Arizona State UniversityTempe, AZ, USA; ^2^The Biodesign Institute, Arizona State UniversityTempe, AZ, USA

**Keywords:** bidirectional hydrogenase, cyanobacteria, *Lyngbya aestuarii*, *Microcoleus chthonoplastes*, *Synechocystis*, hydrogen, fermentation

## Abstract

Cyanobacteria are considered good models for biohydrogen production because they are relatively simple organisms with a demonstrable ability to generate H_2_ under certain physiological conditions. However, most produce only little H_2_, revert readily to H_2_ consumption, and suffer from hydrogenase sensitivity to O_2_. Strains of the cyanobacteria *Lyngbya aestuarii* and *Microcoleus chthonoplastes* obtained from marine intertidal cyanobacterial mats were recently found to display much better H_2_ production potential. Because of their ecological origin in environments that become quickly anoxic in the dark, we hypothesized that this differential ability may have evolved to serve a role in the fermentation of the photosynthate. Here we show that, when forced to ferment internal substrate, these cyanobacteria display desirable characteristics of physiological H_2_ production. Among them, the strain *L. aestuarii* BL J had the fastest specific rates and attained the highest H_2_ concentrations during fermentation of photosynthate, which proceeded via a mixed acid fermentation pathway to yield acetate, ethanol, lactate, H_2_, CO_2_, and pyruvate. Contrary to expectations, the H_2_ yield per mole of glucose was only average compared to that of other cyanobacteria. Thermodynamic analyses point to the use of electron donors more electronegative than NAD(P)H in *Lyngbya* hydrogenases as the basis for its strong H_2_ production ability. In any event, the high specific rates and H_2_ concentrations coupled with the lack of reversibility of the enzyme, at the expense of internal, photosynthetically generated reductants, makes *L. aestuarii* BL J and/or its enzymes, a potentially feasible platform for large-scale H_2_ production.

## Introduction

Cyanobacteria have great potential to act as cell factories, because they have the ability to use light to split water, potentially generating H_2_ (Weaver et al., 1980; Akkerman et al., 2002; Prince and Kheshgi, 2005). They do in fact evolve H_2_ naturally, but as a by-product of N_2_ fixation, or as an end-product of fermentation. Very transitorily, a burst in H_2_ production is sometimes seen when the light is switched on suddenly during dark fermentative metabolism. The latter is the only known form of direct “photohydrogen” production in cyanobacteria. The enzyme responsible for N_2_ fixation, nitrogenase, does also reduce protons and releases H_2_ as an unavoidable side reaction (Peterson and Burris, 1978; Eisbrenner and Evans, 1983). This process requires significant cellular energy inputs and most often does not result in any net H_2_ production, because it is reoxidized via an uptake hydrogenase (Peterson and Burris, 1978). It has been proposed that the enzyme bidirectional hydrogenase is involved in fermentative H_2_ production (Stal and Moezelaar, 1997; Troshina et al., 2002) and photohydrogen generation (Appel et al., 2000). As the name implies this enzyme has the ability to both produce and oxidize H_2_ (Fujita and Myers, 1965). Direct photohydrogen production in cyanobacteria is extremely short-lived (a few seconds) with rather negligible H_2_ yields (Appel et al., 2000). Fermentative H_2_ production represents an indirect hydrophotolytic route that proceeds through an organic intermediary (glycogen). It is relatively long-lived (hours) with somewhat better H_2_ yields than photohydrogen production (Cournac et al., 2002; Troshina et al., 2002). Fermentative H_2_ production is in fact the natural mode by which cyanobacteria release H_2_ for extended periods of time in nature, making it of potential biotechnological interest.

Cyanobacteria have the intrinsic ability to ferment in order to survive dark anaerobic conditions (Gottschalk, 1979). Depending on strain, they have been shown to carry out a variety of fermentative metabolisms including the homolactate, homoacetate, heterolactate, and mixed acid pathways (Stal and Moezelaar, 1997). The homolactate pathway primarily produces lactate (Oren and Shilo, 1979), whereas the heterolactate pathway evolves lactate along with ethanol and acetate (Heyer et al., 1989). The homoacetate pathway produces mostly acetate along with minor quantities of lactate, CO_2_, and H_2_ (Heyer et al., 1989; De Philippis et al., 1996). The mixed acid fermentation pathway is known to produce acetate, lactate, ethanol, formate and/or CO_2_, and H_2_ (Van der Oost et al., 1989; Moezelaar et al., 1996; Aoyama et al., 1997; Troshina et al., 2002). Thus, the mixed acid and, to a certain extent, the homoacetate pathways result in H_2_ production.

Cyanobacteria are not known to respire external electron acceptors other than O_2_, and thus, when subjected to nighttime anoxia must resort to fermentation in order to maintain ATP production and regenerate excess reduction equivalents. A classic example of an environment conducive to this is cyanobacterial benthic mats (Walter, 1976; Bauld, 1981; Javor and Castenholz, 1981). In these mats, oxygenic photosynthetic activity causes the top mat layers to become supersaturated with O_2_ during the daytime, but strong respiration rates overwhelm diffusive O_2_ import in the dark, establishing strong anoxia (Revsbech et al., 1983) and forcing the constituent cyanobacteria to ferment its daytime photosynthate. Fermentation products have been directly detected in hot spring microbial mats (Anderson et al., 1987; Nold and Ward, 1996). Amongst the mat inhabiting cyanobacteria, fermentation has been reported in *Oscillatoria terebriformis* (Richardson and Castenholz, 1987) and *Synechococcus* sp. strains OS-A and OS-B (Steunou et al., 2006) from hot springs. Fermentation has also been studied in marine microbial mat-building *Lyngbya aestuarii* CCY 9616 (= PCC 8106, also known as *Oscillatoria limosa* in the early literature) and *Oscillatoria* sp. SAG 3192 (Garcia-Pichel et al., 1996) [also referred to as *Microcoleus chthonoplastes* 11 or *M. chthonoplastes* SAG 3192 before (Stal and Krumbein, 1985)]. *L. aestuarii* CCY 9616 follows a homoacetate–heterolactate pathway (Heyer et al., 1989) whereas *Oscillatoria* sp. SAG 3192 ferments via a mixed acid fermentation pathway (Moezelaar et al., 1996).

Owing to the presence, multiplicity, and avidity of potential H_2_ consumers in the complex microbial communities where H_2_ is being produced, steady-state concentrations of H_2_ tend to remain very low, usually undetectable in natural systems (Ebert and Brune, 1997; Schink, 1997). This general rule finds a clear exception in some intertidal microbial mats, where net H_2_ accumulation and export has been reported (Skyring et al., 1989; Hoehler et al., 2001). Similarly, it was observed that intertidal microbial mats from Baja California, maintained in a greenhouse setting for more than 3 years under an artificial intertidal regime, continue to produce H_2_ at night, exporting significant amounts to the overlying waters (Hoffmann and Maldonado, personal communication). The organisms fermenting under such conditions must thus be able to produce H_2_ even against high partial pressures in the mat.

In an earlier report surveying a set of cyanobacterial strains for H_2_ production in presence of excess reductants, two different patterns were observed. Pattern 1, known from fresh water strains such as *Synechocystis* sp. PCC 6803, exhibited low rates and steady-state H_2_ concentrations followed by uptake of most of the produced H_2_ whereas the novel Pattern 2, found only in *L. aestuarii* and *M. chthonoplastes* strains from the marine intertidal mats, exhibited much higher rates, steady-state H_2_ concentrations, and a lack of H_2_ uptake throughout the assay (Kothari et al., 2012). Indeed, the cyanobacterial strains isolated from these mats displayed an extraordinary potential to produce/sustain H_2_ under the unusually high concentrations of H_2_ prevailing in their micro-environment. However, all of this was done using standard assays (Kothari et al., 2012) that externally provide excess reductant and anaerobic conditions. Now these studies are extended to include the innate H_2_ evolving capacity under fermentative conditions, using microbiological and genomic evidence.

## Materials and methods

### Strains, media, and growth conditions

Five strains of cyanobacteria were used for this work. *L. aestuarii* BL J, *L. aestuarii* BL AA, and *M. chthonoplastes* BM 003 were isolated from marine intertidal microbial mats in Baja California (Kothari et al., 2012). *M. chthonoplastes* PCC 7420 was originally isolated from a microbial mat in a salt marsh, Woods Hole, Massachusetts.

*Synechocystis* sp. PCC 6803, originally a freshwater isolate, has been used in this study since it is a popular model cyanobacterium for biohydrogen research. The latter two strains were obtained from the Pasteur Culture Collection (http://www.pasteur.fr/ip/easysite/pasteur/en/institut-pasteur). *L. aestuarii* strains were grown in IMR medium with 3% salinity (Eppley et al., 1968), modified to incorporate commercially available Instant Ocean salt mixture instead of natural seawater. *M. chthonoplastes* strains were grown in a 1:1 mixture of IMR and ASN III media (Rippka et al., 1979) with 3% salinity. *Synechocystis* sp. PCC 6803 was grown in BG11 medium (Rippka et al., 1979). All media were supplemented with 0.5 mM (final concentration) NiSO_4_ to ensure adequate supply of nickel for the working of Ni–Fe hydrogenases.

The strains *L. aestuarii* BL J, *L. aestuarii* BL AA, and *M. chthonoplastes* BM 003 were clonal and monocyanobacterial, but not always axenic. Therefore, phase contrast microscopy was used to confirm that the level of contaminating bacteria was less than 0.01% of the cyanobacterial biomass (assessed as bio-volume) for the physiological experiments. *M. chthonoplastes* PCC 7420 and *Synechocystis* sp. PCC 6803 were always used in axenic form.

For the purpose of whole genome sequencing, an axenic culture of *L. aestuarii* BL J was established by picking up the motile hormogonia developing on IMR medium—1% nobel agar plates (Rippka, 1988). These hormogonia were allowed to grow on IMR–PGY medium 1% nobel agar plates (0.25% peptone, 0.25% yeast extract, 0.25% glucose, 1.5% agar), and axenicity was determined by lack of heterotrophic bacterial growth, and through direct microscopic observation. All strains were maintained in 250 ml Erlenmeyer flasks, with 100 ml medium, starting with similar amounts of inoculum in presence of light at an intensity of 100 μmol photon m^−2^ s^−1^.

### Fermentative H_2_ production assay

All strains were subjected to two different sets of growth conditions for the fermentation assays. In the first set, filaments were grown in continuous light without any bubbling (CL). In the second set, the strains were grown in 12-h light and 12-h dark cycle. The cultures were bubbled with air in the light period and with N_2_ in the dark period to establish anoxia, forcing cells to ferment. These conditions are referred to as “Light Oxic Dark Anoxic” (L_O_D_A_) conditions. All cultures were incubated for a minimum of two weeks before making any measurements. The assay itself was carried out in the dark using whole cells (*in vivo*) without the addition of any external reductants. Particularly, for the cultures growing in L_O_D_A_ conditions, the assay was commenced at the beginning of the dark period. Small pea size pellets of biomass from log phase cultures were placed in a custom-made, 2.5 ml volume chamber with continuous stirring. Fresh medium was added to completely fill the chamber, which was sealed with no headspace. The chamber was endowed with two miniature Clark-type electrodes to monitor H_2_ and O_2_ partial pressure. The electrodes were connected to a pico-ammeter set at a voltage of 0.8 V for H_2_ and -0.8 V for O_2_. An A/D converter allowed the current signal data to be read on a computer using Sensor Trace Basic software. All electrodes and peripherals were from Unisense, Aarhus, Denmark. Before each measurement, the H_2_ electrode was subject to a two-point calibration in culture medium bubbled with either air (0% H_2_) or with a custom gas mixture (10% H_2_ in N_2_). The O_2_ electrode was also subjected to a two-point calibration system wherein culture medium was bubbled with either air (21% O_2_) or with 100% N_2_ (0% O_2_). During calibration the sealed chamber showed negligible leakage over a period of 2–3 h.

Each strain was measured in independent triplicate experiments. From the electrode traces, the following parameters were derived: the initial specific rate of fermentative H_2_ production, *R*_*H*_; the maximum steady-state H_2_ concentration reached, *[H*_2_*]_M_*; and the time after which H_2_ production stopped and reverted to consumption, *T*_*R*_. The measurements lasted for 24 h. At the end of the assay, chlorophyll was extracted from the biomass with 100% methanol and measured spectrophotometrically (MacKinney, 1941). This was done to ensure that all assays had roughly comparable biomass and to obtain specific rates of initial H_2_ production (i.e., per unit biomass).

### Analysis of fermentation metabolism in *L. aestuarii* BL J

*L. aestuarii* BL J that had been grown in L_O_D_A_ conditions from three different flasks (replicates) were used in this assay. A couple of hours before the onset of a dark anaerobic period, biomass was harvested by centrifugation, acclimatized to fresh medium, and washed twice with fresh medium to get rid of any potentially existing fermentation products. The filaments form tight clumps, and hence, attempts were made to break the clumps using forceps and mild sonication at the lowest speed setting for 4 s to get a homogeneous cell suspension. This was required to split the biomass into two aliquots with approximately equal amounts of biomass to conduct the initial and final analyses quantifying the fermentation substrates and products. Since a non-destructive procedure that does not impart any kind of stress to the cells was necessary for quantifying the biomass, wet weights were used. Optical density cannot be employed for biomass estimation given the filamentous and clumpy nature of this strain. As described below, since only the wet weights from the two halves of the same filter were compared to each other, the errors in biomass estimation were minimized.

For obtaining two aliquots with approximately equal amounts of biomass using wet weights, the following procedure was adopted. The biomass was vacuum filtered onto a 0.4 μm polycarbonate filter to establish a homogenous layer on it. The filter was cut into half, biomass scrapped off, and the wet weight of the cells on each half was measured. The biomass from each half of the filter was then introduced into a 10 ml serum bottle (one for initial and one for final analyses). To each bottle 5 ml of fresh medium was added and the bottles were sealed. To confirm the initial absence of fermentation products, 1 ml of medium was drawn out from the “initial” serum bottle for later High Pressure Liquid Chromatography (HPLC) analysis. The rest was immediately frozen in liquid N_2_ and stored at −80°C to be used eventually in determining the initial fermentable glycogen content in the cells. The “final” serum bottle was bubbled with nitrogen for 30 min to establish anoxia. Gas Chromatography (GC) confirmed the absence of O_2_, and the serum bottle was incubated in the dark on a rocking bench for 24 h at 25°C.

After incubation, 1 ml of medium was withdrawn for HPLC analyses of organic acids and ethanol. Hydrochloric acid was then added to the serum bottle to lower the pH of the solution and ensure that all the inorganic carbon was present as CO_2_. The gases in the headspace (CO_2_, H_2_, and/or O_2_) were sampled by syringe and quantified by GC equipped with a thermal conductivity detector. GC was performed with Helium as the carrier gas and the concentrations of H_2_ and CO_2_ in the headspace were estimated as described before (Parameswaran et al., 2009). Total masses of gases were back-calculated according to volumetric partitioning. The contents of the serum bottle were then frozen in liquid N_2_ and kept at −80°C for eventual glycogen content quantification.

Glycogen was extracted as per Ernst et al. (1984) and quantified using a BioAssay Systems glycogen assay kit. To determine organic acids and ethanol, HPLC was employed. All the liquid samples for HPLC were filtered through a 0.2 mm PVDF filter and the filtrate used. The HPLC was performed with Aminex HPX–87H column at 50°C with 2.5 mM sulfuric acid as eluent at a flow rate of 0.6 ml/min using a photodiode array and refractive index indicator (Parameswaran et al., 2009). Most of the common products of bacterial fermentation can be detected under these settings.

### Genomic and bioinformatics analyses

The genomic DNA preparation of *L. aestuarii* BL J was subjected to MiSeq 250 Illumina sequencing, assembly, and annotation (Kothari et al., 2013). The genomic sequence of *L. aestuarii* BL J was checked for the presence of orthologs of genes potentially coding for key enzymes involved in fermentation. Protein sequences coding for cyanobacterial fermentation enzymes from NCBI database were used as query and Psi BLAST was performed against the entire *L. aestuarii* BL J genome. Given that the genome is not closed, the absence of any one gene does not necessarily imply its absence from the genome, as there is a small probability that it is found in unsequenced regions. This Whole Genome Shotgun project has been deposited at DDBJ/EMBL/GenBank under the accession AUZM00000000. The version described in this paper is version AUZM01000000.

## Results

### Fermentative H_2_ production

The strains *L. aestuarii* BL J, *L. aestuarii* BL AA, *M. chthonoplastes* BM 003, *M. chthonoplastes* PCC 7420, and *Synechocystis* sp. PCC 6803 were all capable of fermentative H_2_ production. All strains reached anoxic conditions solely by dark respiration without the addition of any external reductants, anoxia-inducing compounds, or fermentable substrates. As soon as anoxia was established, H_2_ production commenced without any measurable lag time in all strains.

**Figure 1:**
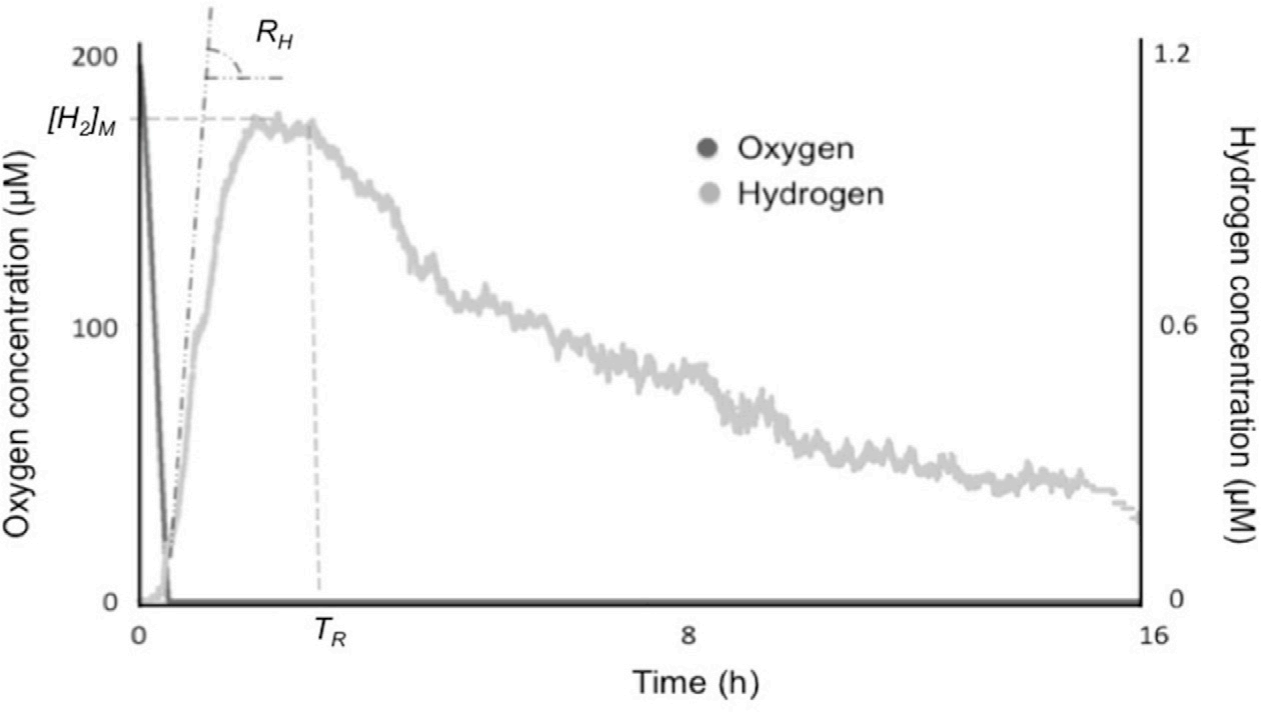
**Oxygen and hydrogen concentrations during a fermentative H_2_ production assay in *Synechocystis* sp. PCC 6803**. Anoxia is established in a few minutes by respiration in dark followed by onset of fermentative H_2_ production. The parameters of H_2_ production studied are the maximal initial rate of H_2_ production, *R*_*H*_, the maximum steady-state H_2_ concentration *[H*_2_*]_M_*, and the time, after which hydrogenase reverses in direction, *T*_*R*_.

Some variation in the parameters of fermentative H_2_ production could be detected. These main parameters are initial specific rate, *R*_*H*_, the maximum H_2_ steady-state concentration, *[H*_2_*]_M_*, and the time after which the enzyme reverts in direction, *T*_*R*_ (Figure 1). Table 1 gathers information on these parameters for all the five tested strains. In general, *Lyngbya* and *Microcoleus* strains from microbial mats produced H_2_ faster and could reach higher equilibrium concentrations of H_2_ than the standard strain *Synechocystis* sp. PCC 6803. *Lyngbya* and *Microcoleus* strains did not consume the H_2_ produced during the assay (for up to 24 h) unlike *Synechocystis* sp. PCC 6803. The highest specific rate of H_2_ production was seen in *L. aestuarii* BL AA and the highest steady-state H_2_ concentration was seen in *M. chthonoplastes* BM 003.

**Table 1:** **Parameters characterizing the dynamics of fermentative H_2_ production in various cyanobacterial strains along with the effect of prior exposure to nighttime anoxia**.

**Strain**	**Growth condition**	***R_H_* [nmol (μg chl. *a*)^1^h^1^]**	***[H_2_]_M_* (μM)**	***T*_*R*_ (h)**
*L. aestuarii* BL J	L_O_D_A_	5.3 ± 2.7	159.8 ± 30.4	2:24
	CL	2.8 ± 1.7	5.6 ± 4.0	>24
*L. aestuarii* BL AA	L_O_D_A_	4.0 ± 2.9	87.8 ± 43.0	2:24
	CL	9.4 ± 0.1	4.70 ± 2.9	>24
*M. chthonoplastes* PCC 7420	L_O_D_A_	0.4 ± 0.4	2.3 ± 0.6	2:24
	CL	0.4 ± 0.2	2.6 ± 1.2	>24
*M. chthonoplastes* BM 003	L_O_D_A_	0.6 ± 0.1	2.7 ± 1.5	2:24
	CL	0.8 ± 0.1	35.5 ± 17.4	>24
*Synechocystis* sp. PCC 6803	L_O_D_A_	0.3 ± 0.2	3.5 ± 2.8	5 ± 6.6
	CL	0.2 ± 0.1	2.7 ± 2.0	3.3 ± 3.2

Averages ± standard deviation with n = 3 independent determinations shown for each strain and condition. CL, Continuous Light; L_O_D_A_, Light Oxic and Dark Anoxic, 12-h cycles.

### Optimization of fermentative H_2_ production

**Figure 2:**
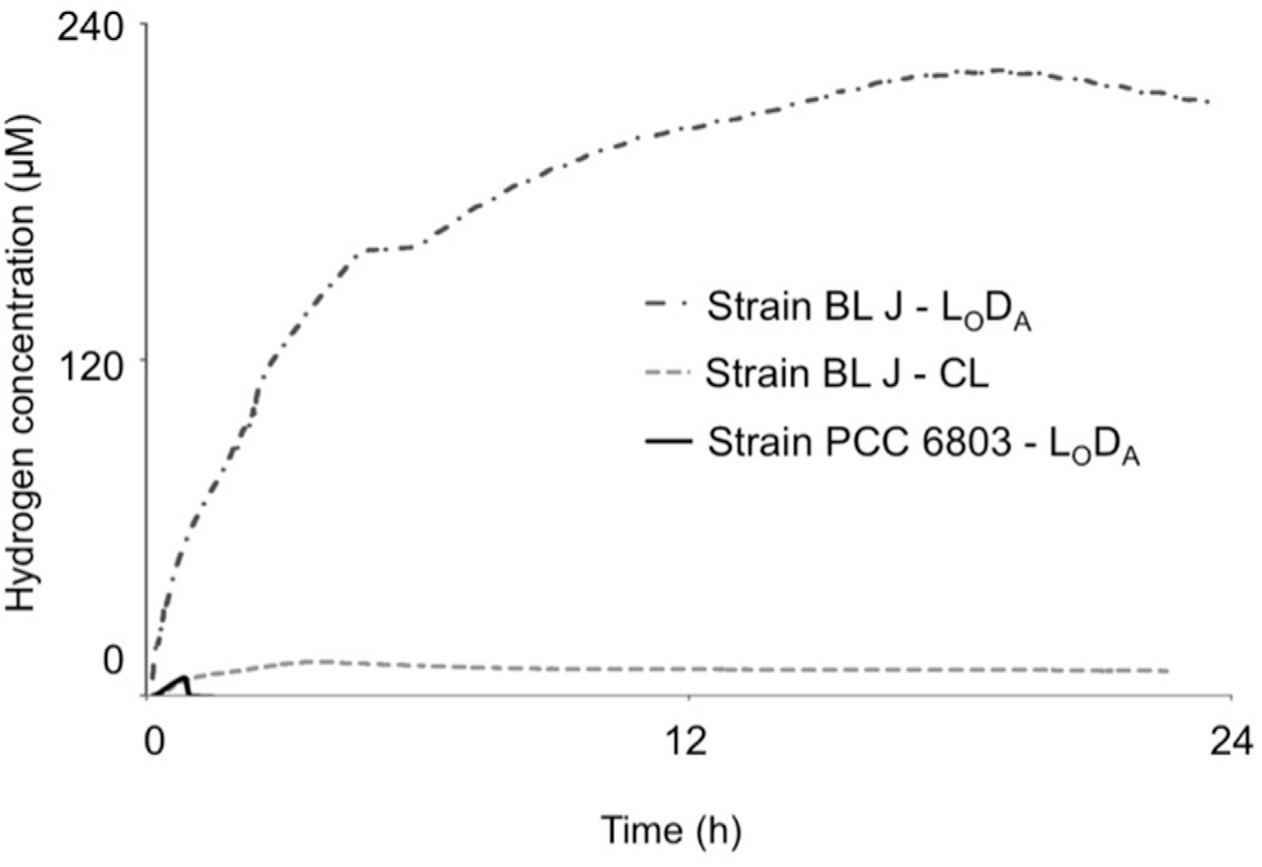
**Comparison of the dynamics of fermentative hydrogen production in continuous light (CL) grown *L. aestuarii* BL J, along with Light Oxic Dark Anoxic (L_O_D_A_) cycle grown *L. aestuarii* BL J and *Synechocystis* sp. PCC 6803**.

Attempts were made to optimize the H_2_ produced by acclimatizing the cells to 12-h light/dark cycles wherein the cells were exposed to anoxia in the dark (L_O_D_A_). *Synechocystis* sp. PCC 6803 showed no significant improvements by this preconditioning in any of the parameters. The specific rates and steady-state concentrations of fermentative H_2_ production attained in *Lyngbya* strains, but not those of *Microcoleus* strains, could be enhanced when cultures were pre-acclimated to recurrent nighttime anaerobiosis during growth. On subjecting the strains to L_O_D_A_ preconditioning all strains retained their characteristic feature of reversibility of reaction direction (or the lack of it). *L. aestuarii* BL J reached the highest specific rates and steady-state concentrations of H_2_. The *R*_*H*_ of *L. aestuarii* BL J grown in L_O_D_A_ condition doubled compared to that of cells grown in continuous light conditions; its *[H*_2_*]_M_* increased 28-fold (Figure 2). In L_O_D_A_ conditions, the strain BL J performed exceptionally better than the standard *Synechocystis* sp. PCC 6803, [its *R*_*H*_ was 20-fold faster and *[H*_2_*]_M_* 45-fold higher (Figure 2)]. While calculating the average *R*_*H*_ of *L. aestuarii* BL J grown in L_O_D_A_ one abnormally high specific rate of 44.2 nmol (μg chl. *a*)^−1^h^−1^ was removed from the tally. Had this been incorporated, the *R*_*H*_ value would have been 13.1 ± 17.5 nmol (μg chl. *a*)^−1^h^−1^.

Attempts made to further optimize the fermentative H_2_ production from *L. aestuarii* BL J in L_O_D_A_ conditions by varying the salinity, nickel and nitrate content in the medium did not lead to any significant increase in the specific rates or steady-state H_2_ concentrations (data not shown). On starving cells of nickel, however, a 15-fold decrease in the specific rates of fermentative H_2_ production was observed, indicating the nickel dependency of the enzyme system involved in the process.

### Fermentation in *L. aestuarii* BL J

Since *L. aestuarii* BL J displayed the highest *R*_*H*_ and *[H*_2_*]_M_*, it was chosen for further analysis. Along with H_2_, fermentative production of lactate, ethanol, acetate, and CO_2_ was observed. The ratio of the products of fermentation remained similar for the three independent replicate experiments. Small amounts of pyruvate were also excreted. Other common bacterial fermentation products such as formate, succinate, propionate, and butyrate were not detected. Table 2 depicts a quantitative balance analysis of the fermentation process in *L. aestuarii* BL J. Ethanol and acetate were produced in equimolar amounts. Lactate, ethanol, and acetate were seen in 1:2:2 molar ratios. One mol of H_2_ was produced for every 2 moles of CO_2_. In our experiments, the stoichiometry of carbon recovery and the recovery of H available was 100.07 and 100.58%, respectively.

**Table 2:** **Stoichiometry of fermentation of endogenous polyglucose and the fermentation mass balance of *L. aestuarii* strain BL J, after 24 h of dark incubation**.

	**mol**	**mol/100 mol glucose**	**mol C/100 mol glucose**	**H available**	**H available mol/100 mol glucose**
**PRODUCTS**					
Glucose	13.27	100.00	600.00	24.00	2400.00
Pyruvate	0.46	3.48	10.44	10.00	34.81
Lactate	5.91	44.54	133.61	12.00	534.44
Acetate	11.44	86.21	172.41	8.00	689.66
Ethanol	11.34	85.48	170.95	12.00	1025.72
H_2_	8.37	63.07	0.00	2.00	126.15
C0_2_	113.04	113.04	113.04	0.00	0.00
Recovery (%)			100.07		100.58

H available is determined by oxidizing a compound to carbon dioxide with water. For example, C_6_H_12_O_6_ + 6H_2_O → 24H + 6CO_2_. Thus, the H available value for glucose is 24 (Gottschalk, 1979).

### Genomic evidence

Based on the whole genome sequence of the strain BL J (Kothari et al., 2013), orthologs of the following genes involved in glycogen metabolism were detected: Glucose-1-phosphate adenylyltransferase, glycogen synthase, ADP-glucose transglucosylase, glycogen branching enzyme (GH-57-type, archaeal), 1,4-alpha-glucan (glycogen) branching enzyme (GH-13-type), glycogen debranching enzyme, glycogen phosphorylase, and 4-alpha-glucanotransferase (amylomaltase). Orthologs of genes coding for all the enzymes involved in the pentose phosphate pathway and glycolysis, potential routes for the breakdown of glucose into pyruvate, were detected.

**Figure 3:**
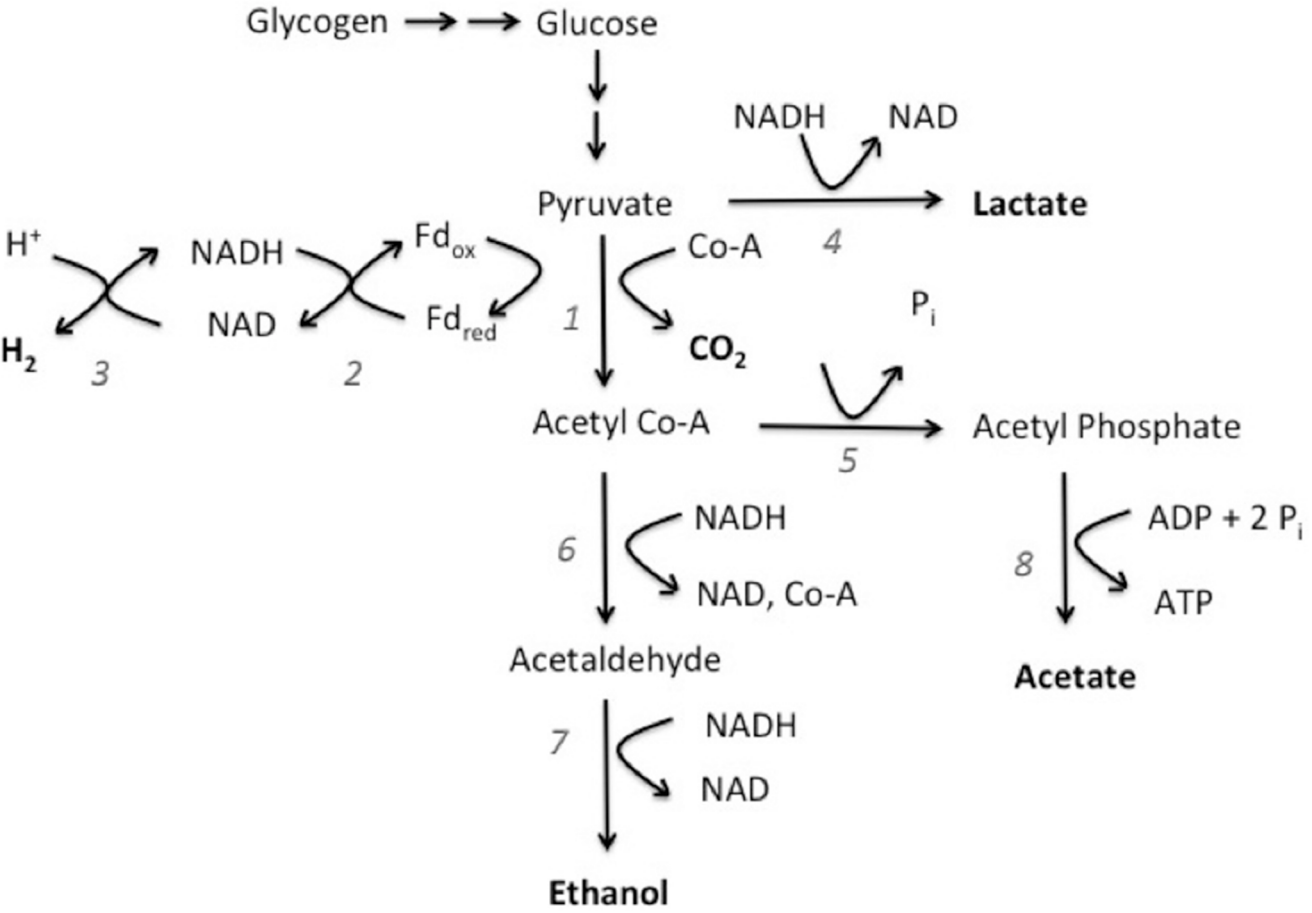
**Proposed fermentation pathway of glycogen (polyglucose) fermentation in *L. aestuarii* BL J**. Compounds in bold are fermentation products. The numbers refer to the enzymes involved: 1, pyruvate ferredoxin oxidoreductase; 2, ferredoxin NADP oxidoreductase; 3, bidirectional hydrogenase; 4, lactate dehydrogenase; 5, phosphotransacetylase; 6, acetaldehyde dehydrogenase; 7, alcohol dehydrogenase; 8, acetate kinase.

Genomic evidence for the presence of mixed acid fermentation pathway was clear. *L. aestuarii* BL J has orthologs coding for the enzymes pyruvate:ferredoxin oxidoreductase, ferredoxin:NADP oxidoreductase, bidirectional hydrogenase, lactate dehydrogenase, phosphotransacetylase, acetaldehyde dehydrogenase, alcohol dehydrogenase, and acetate kinase (see supplementary information for gene accession numbers). Based on the fermentation products obtained experimentally and the presence of these orthologs, the pathway proposed for fermentative degradation of glycogen is depicted in Figure 3. Notably, the gene for pyruvate formate lyase, involved in the reversible conversion of pyruvate and coenzyme-A into formate and acetyl-CoA, was not detected. This was consistent with a lack of formate amongst the fermentation products. Also worth noting is that no genomic evidence could be found for formate hydrogen lyase, involved in splitting of formate into H_2_ and CO_2_.

It was of interest to positively identify the enzyme responsible for the intense H_2_ evolution observed in this study, as this may be the target of future studies. H_2_ production in fermentative pathways can be either of *Enteric* type, with H_2_ evolving from formate breakdown by formate hydrogen lyase, or of *Clostridial* type, wherein H_2_ is evolved by pyruvate:ferredoxin oxidoreductase along with a hydrogenase (Hallenbeck, 2009). Based on the genome, *L. aestuarii* BL J lacks both enzymes for the *Enteric* pathway, but contains a pyruvate:ferredoxin oxidoreductase and two [Ni–Fe] hydrogenases, an uptake-type hydrogenase and a bidirectional-type hydrogenase. One of these two must be involved, given that fermentative H_2_ production in *L. aestuarii* BL J was found to be Ni-dependent. Because uptake hydrogenases are not known to produce H_2_ under physiological conditions (Houchins and Burris, 1981; Houchins, 1984) we propose that the Ni–Fe bidirectional hydrogenase is the source of the fermentative H_2_ produced in *L. aestuarii* BL J.

The strain BL J contains homologs of genes coding for nitrogenase, an enzyme that could in theory contribute to H_2_ production in this strain. However, since the fermentation assays were performed in presence of nitrate, a condition in which the nitrogenase is known to be inactive (Ferreira, 2009), the role of the enzyme in production of H_2_ is ruled out. The homologs of genes coding for uptake hydrogenase are also present in the strain BL J. However, the uptake hydrogenase is not capable of H_2_ production in physiological conditions; in fact, it has to be knocked out to attain considerable H_2_production via the nitrogenase (Lindberg et al., 2002; Lindblad et al., 2002; Masukawa et al., 2002; Yoshino et al., 2007) and also via the fermentation pathway (Kim et al., 2006; Zhao et al., 2009).

## Discussion

### The physiological basis for strong fermentative hydrogen production

We had previously demonstrated that in standard H_2_ production assays, strains of *Lyngbya* and *Microcoleus* displayed optimal H_2_ evolution characteristics compared to a large number of other strains from diverse environments (Kothari et al., 2012). Since excess reductants were externally provided during that assay, the results likely are maximal potential specific rates and do not actually reflect physiologically realistic conditions. This prompted us to study the actual H_2_ production capacity of these strains. Here we demonstrate that all the four strains studied had the capacity to produce fermentative H_2_ naturally, at the expense of photosynthetically fixed carbon, as did the standard strain *Synechocystis* sp. PCC 6803, which is included for reference. As reported earlier (Cournac et al., 2002), the fermentative H_2_ evolution in *Synechocystis* sp. PCC 6803 commenced without any lag time, as was the case in *Microcoleus* and *Lyngbya* strains, and unlike what was observed in *Microcystis aeruginosa* M-176 (Asada and Kawamura, 1984). In general, the specific H_2_ production rates and the steady-state concentrations under fermentative conditions were about an order of magnitude lower than the potential seen in standard assays in presence of excess reductant (Kothari et al., 2012). The *Microcoleus* and *Lyngbya* strains from the marine intertidal mats are capable of sustained fermentative H_2_ production for at least 24 h. This was in accordance to the Pattern 2 H_2_ production earlier reported in these strains via the hydrogenase activity assay wherein sustained H_2_ production was also measured for up to 24 h. In comparison, the H_2_ production phase did not last more than about 3 h in the standard strain *Synechocystis* sp. PCC 6803 (in agreement with Pattern 1 H_2_ production earlier reported in this strain). This is consistent with the notion that cyanobacteria isolated from environments experiencing recurring nighttime anoxia (marine microbial mats) may be innately better H_2_ producers, thus validating a general approach of bio-prospecting in nature for biotechnologically useful properties of extant but little known microbes.

That H_2_ production metabolism in intertidal mat harboring *Lyngbya* strains was enhanced by prior exposure to recurrent dark anaerobic growth conditions was expected under the premise that this type of fermentative metabolism would be regulated and thus subject to induction. This was clearly not the case in *Microcoleus* strains, where the capacity for fermentative H_2_ generation, while high, seemed to be constitutive. *Lyngbya* typically colonizes microbial mats that desiccate frequently and are not exposed to nighttime anoxia as frequently as *Microcoleus*, which tends to dominate mats lower in the tidal gradient, with more recurrent flooding or always flooded (Javor and Castenholz, 1981; Rothrock and Garcia-Pichel, 2005). Perhaps the different responsiveness of the fermentative H_2_ physiology has to do with this differential ecology. *Synechocystis* sp. PCC 6803, which has been in culture since 1968 does presumably not see many periods of dark anoxia during cultivation, and displayed a low-yield, non-inducible H_2_ physiology.

When forced to ferment on a diel cycle, the highest specific rate and steady-state H_2_ concentration was exhibited by *L. aestuarii* BL J. In comparison, to *Synechocystis* sp. PCC 6803, the initial rates of H_2_ production were 17-fold higher in *L. aestuarii* BL J in the optimized fermentation assays. Most likely, this is due to increased content of bidirectional hydrogenase in the strain BL J or because *Synechocystis* sp. PCC 6803 employed alternative strategies in dark anaerobic conditions to regenerate NAD(P)^+^ or because the Michaelis constant (K_M_) of the bidirectional hydrogenase in the strain BL J is more favorable for H_2_ production.

The major difference between *L. aestuarii* BL J and *Synechocystis* sp. PCC 6803 was that the latter exhibited a decline in the concentrations of H_2_ leading to the consumption of almost all the H_2_ produced. This decline was seen in presence of excess external reductants (Kothari et al., 2012) and internal reductants (Figure 1) implying that the concentration of reductants was not the limiting factor. The observed decline also has little to do with the loss of enzyme activity, since the bidirectional hydrogenase works in the direction of H_2_ consumption and is thus still active. We must thus postulate a regulatory cause for these differences.

The optimized steady-state H_2_ concentrations in fermentative assays in the strain BL J were only three-fold lower in magnitude than those seen in standard assays in the presence of excess reductant (Kothari et al., 2012). This is suggestive of the presence of a strong H_2_ producing system, which, might be of particular fitness value in the uniquely H_2_ accumulating intertidal mats. In optimized fermentation assays, the steady-state H_2_ concentration in the strain BL J was 45-fold higher than that reached by *Synechocystis* sp. PCC 6803. Free sugar concentrations during fermentation are unlikely to be so different between the two cyanobacteria to account for such differences, and, even in the presence of excess externally provided reductants, the steady-state H_2_ concentration in *Synechocystis* sp. PCC 6803 was 15-fold lower than the strain BL J (Kothari et al., 2012). Again here, the only possible explanation for this behavior is some sort of regulation of the hydrogenase enzyme in the strain PCC 6803.

On the basis of thermodynamics it may be theoretically possible (albeit difficult) for NAD(P)H to act as the sole electron donor to the bidirectional hydrogenase in *Synechocystis* sp. PCC 6803 during the fermentation assay, The intracellular ratio of [NAD(P)H] to [NAD(P)^+^] required for *Synechocystis* sp. PCC 6803 to produce 3 μM H_2_ at equilibrium is 7.63. In *Synechocystis* sp. PCC 6803, the measured [NADPH]/[NADP^+^] is 3.03 under light oxic conditions (Cooley and Vermaas, 2001), but in dark anaerobic conditions the cell is even more reduced, so the ratios are expected to be higher. This is not unlike the ratios found in heterotrophic bacteria, which can go up to 6.66–0.7 (Decker and Pfitzer, 1972; Lee et al., 2009; Siedler et al., 2011). However, the [NAD(P)H]/[NAD(P)^+^] theoretically required to produce 150 μM H_2_ at equilibrium, like *L. aestuarii* BL J does, is 385. It is highly unlikely such ration can be achieved by the cell and hence it is implausible for NAD(P)H to act as the sole electron donor to the bidirectional hydrogenase in *L. aestuarii* BL J during the fermentation assay.

We propose that the *Lyngbya* can make so much H_2_ because they efficiently use more electronegative electron donors for the bidirectional hydrogenase than NAD(P)H. Flavin adenine dinucleotide (FAD) (−0.219 to −0.400 V) (Nelson et al., 2008; Faro et al., 2002), thioredoxin (−0.200 to −0.350 V) (Krause et al., 1991) and ferredoxin (−0.432 V) (Nelson et al., 2008), could all act as potential electron donors for the bidirectional hydrogenase enzyme. In fact, a recent study (Gutekunst et al., 2014) proposed that the bidirectional hydrogenase mediated H_2_ production in *Synechocystis* sp. PCC 6803 is coupled to ferredoxin and flavodoxin. If this is indeed true, it is of interest to speculate why the diaphorase subunit [involved in interactions with NAD(P)H and NAD(P)^+^] is associated with bidirectional hydrogenase in most cyanobacterial strains. Perhaps the main role of the diaphorase is in the oxidation of H_2_, to regenerate NAD(P)H.

As an aside and a caveat, if the intracellular pH of the strain BL J was as low as five, that would bring the required [NAD(P)H]/[NAD(P)^+^] to 3.8, making it feasible to attain high H_2_ concentrations using NAD(P)H as an electron donor. However, such intracellular pH would be unprecedented, particularly given the extreme sensitivity of cyanobacteria to even moderately acidic conditions.

### Fermentation pathways among the filamentous non-heterocystous cyanobacteria

*L. aestuarii* BL J has all the products and the orthologs of genes coding for all enzymes of the mixed acid fermentation pathway. In view of the stoichiometric ratios of the products of fermentation (Table 2), our strain does not follow any one ideal fermentation pathway or even a combination of pathways. This is also the case for *Cyanothece* sp. PCC 7822 (Van der Oost et al., 1989), *Microcystis* sp. PCC 7806 (Moezelaar and Stal, 1994), and *Oscillatoria* sp. SAG 3192 (Moezelaar et al., 1996). The fermentation pathway is likely to be similar to that observed in *Microcystis* sp. PCC 7806 (Moezelaar and Stal, 1994) and *Gloeocapsa alpicola* CALU 743 (Troshina et al., 2002).

Since *L. aestuarii* BL J accumulates very high concentrations of H_2_, it was of significance to characterize the molar conversion ratio of glucose to H_2_. Theoretically 1 mole of glucose can give rise to a maximum of 4 moles of H_2_ via fermentation (Thauer, 1977). Amongst cyanobacteria, this theoretical maximum has been observed only in *G. alpicola* CALU 743 (Troshina et al., 2002). *Microcystis* sp. PCC 7806 yields 0.51 (Moezelaar and Stal, 1994) and *Cyanothece* sp. PCC 7822 yields 0.76 moles/mol (Van der Oost et al., 1989). *L. aestuarii* BL J, at 0.6 moles/mol, was certainly not the best. The strong H_2_ production characteristics of *L. aestuarii* BL J cannot be attributed to a high glucose to H_2_ molar conversion ratio, but likely reside in the bidirectional hydrogenase enzyme system.

It may be of interest to discuss the fermentative metabolism of strain BL J in comparison to the few other closely related cyanobacterial species whose fermentation has been studied in any detail. *L. aestuarii* CCY 9616, a strain phylogenetically close to *L. aestuarii* BL J (99% identity based on 16S rRNA), behaves quite differently: it can ferment trehalose, its osmoprotectant, via the homoacetate pathway into mostly acetate with small amounts of H_2_ and CO_2_ (Heyer et al., 1989). It can also ferment glycogen by a heterolactic fermentation pathway producing equimolar amounts of ethanol, lactate, and CO_2_ (Heyer et al., 1989). It is unknown if *L. aestuarii* BL J has the same osmoprotectant, or if it can be metabolized via a similar fermentation. This is however unlikely, in that neither the enzyme trehalase, involved in trehalose breakdown, nor the carbon monoxide dehydrogenase, a key enzyme for homoacetic fermentation, could be detected in its genome. Notably, the genome had orthologs of the enzymes trehalose synthase and trehalose-6-phosphate synthetase involved in trehalose synthesis. This may be worth a direct assessment. Another closely related strain, the thermophilic *O. terebriformis*, was also fermentatively distinct; it produced lactate by anaerobic degradation of photosynthetically accumulated glycogen, without producing acetate, butyrate, isobutyrate or n-butyrate (Richardson and Castenholz, 1987). In comparison, *L. aestuarii* BL J produces equimolar amounts of ethanol and acetate, a characteristic feature also seen in more distantly related *Oscillatoria* sp. SAG 3192, which also uses the mixed acid fermentation pathway (Moezelaar et al., 1996). Phylogeny seems thus to be a poor predictor of fermentative pathways.

In conclusion, the H_2_ production in the strain BL J is unique in accumulating high steady-state concentrations of H_2_ thus simplifying harvest of the end product and making it desirable for long-term applications.

## Author contributions

Concept by Ankita Kothari and Ferran Garcia-Pichel, all the experimental work and analysis done by Ankita Kothari and with assistance from Prathap Parameswaran on HPLC analysis. Writing by Ankita Kothari and editorial help by Ferran Garcia-Pichel.

### Conflict of interest statement

The authors declare that the research was conducted in the absence of any commercial or financial relationships that could be construed as a potential conflict of interest.
