# Supplementary material for: Powerful fermentative hydrogen evolution of photosynthate in the cyanobacterium Lyngbya aestuarii BL J mediated by a bidirectional hydrogenase
Source: Front Microbiol. 2014 Dec 10;5:680. doi: 10.3389/fmicb.2014.00680 (PMC4261827; doi:10.3389/fmicb.2014.00680)
Supplement: Supplementary file 1 [file Table1.DOCX]

Supplementary Information: Accession numbers of homologs of genes involved in mixed acid fermentation in *Lyngbya aestuarii* BL J, along with its amino acid identity to the closest gene allele with tested gene activity.

| Enzyme name | Accession Number in BL J | Accession number of the closest gene allele with tested gene activity | % Amino Acid Identity | Reference |
| --- | --- | --- | --- | --- |
| Pyruvate ferredoxin oxidoreductase | [WP_023068000.1](http://www.ncbi.nlm.nih.gov/protein/553733355?report=genbank&log$=prottop&blast_rank=2&RID=5JT44VA9013) | *Lyngbya* sp. PCC 8106 (WP_009785511.1) | 94 | (Heyer et al., 1989) |
| Ferredoxin NADP oxidoreductase | [WP_023069632.1](http://www.ncbi.nlm.nih.gov/protein/553735062?report=genbank&log$=prottop&blast_rank=13&RID=5JTKB41Z013) | *Nostoc* sp. 7119 ([P21890.2](http://www.ncbi.nlm.nih.gov/protein/585127?report=genbank&log$=prottop&blast_rank=1&RID=5JWXJD3D015)) | 60 | (Walker et al., 1990) |
| Lactate dehydrogenase | [WP_023065725.1](http://www.ncbi.nlm.nih.gov/protein/553731045?report=genbank&log$=prottop&blast_rank=2&RID=5JTCKN3K013) | *Lyngbya* sp. PCC 8106 ([WP_009783467.1](http://www.ncbi.nlm.nih.gov/protein/497469269?report=genbank&log$=prottop&blast_rank=1&RID=5JTCKN3K013)) | 94 | (Heyer et al., 1989) |
| Phosphotrans-acetylase | [WP_023069760.1](http://www.ncbi.nlm.nih.gov/protein/553735213?report=genbank&log$=prottop&blast_rank=175&RID=5JXZVGCV013) | *Synechocystis* sp. PCC 6803  ([WP_010872337.1](http://www.ncbi.nlm.nih.gov/protein/499174750?report=genbank&log$=prottop&blast_rank=1&RID=5JXU50S2013)) | 47 | (Juntarajumnong et al., 2007) |
| Acetaldehyde dehydrogenase | [WP_023068380.1](http://www.ncbi.nlm.nih.gov/protein/553733745?report=genbank&log$=prottop&blast_rank=1&RID=5JS4710N015) | *Microcystis aeroginosa* PCC 7806 (CAO87569.1) | 47 | (Moezelaar and Stal, 1994) |
| Alcohol dehydrogenase-1 | [WP_023068380.1](http://www.ncbi.nlm.nih.gov/protein/553733745?report=genbank&log$=prottop&blast_rank=1&RID=5JS4710N015) | *Lyngbya* sp. PCC 8106  ([WP_009785271.1](http://www.ncbi.nlm.nih.gov/protein/497471073?report=genbank&log$=prottop&blast_rank=2&RID=5JS4710N015)) | 47 | (Heyer et al., 1989) |
| Alcohol dehydrogenase -2 | [WP_023064396.1](http://www.ncbi.nlm.nih.gov/protein/553729705?report=genbank&log$=prottop&blast_rank=1&RID=5UF0CNXK015) | *Lyngbya* sp. PCC 8106 ([WP_009783975.1](http://www.ncbi.nlm.nih.gov/protein/497469777?report=genbank&log$=prottop&blast_rank=5&RID=5UG86NTK013)) | 98 | (Heyer et al., 1989) |
| Acetate kinase | [WP_023068105.1](http://www.ncbi.nlm.nih.gov/protein/553733463?report=genbank&log$=prottop&blast_rank=1&RID=5JRVS88K013) | *Lyngbya* sp. PCC 8106  ([WP_009785197.1](http://www.ncbi.nlm.nih.gov/protein/497470999?report=genbank&log$=prottop&blast_rank=2&RID=5JRVS88K013)) | 94 | (Heyer et al., 1989) |
| Bidirectional hydrogenase | [WP_023067990.1](http://www.ncbi.nlm.nih.gov/protein/553733345?report=genbank&log$=prottop&blast_rank=3&RID=5JUU2P1C015) | *-* | - | (Kothari et al., 2012) |
